# Advanced integration of fluid dynamics and photosynthetic reaction kinetics for microalgae culture systems

**DOI:** 10.1186/s12918-018-0611-9

**Published:** 2018-11-20

**Authors:** Stepan Papacek, Jiri Jablonsky, Karel Petera

**Affiliations:** 1 Institute of Complex Systems, South Bohemian Research Center of Aquaculture and Biodiversity of Hydrocenoses, Faculty of Fisheries and Protection of Waters, University of South Bohemia in České Budějovice, Zámek 136, 373 33 Nové Hrady, Czech Republic; 2 Czech Technical University in Prague, Faculty of Mechanical Engineering, Technická 4, Prague, 160 00 Czech Republic

**Keywords:** Microalgae, Mathematical modeling, Photosynthesis, CFD, Microalgae culture systems, Flashing light enhancement

## Abstract

**Background:**

Photosynthetic microalgae have been in the spotlight of biotechnological production (biofuels, lipids, etc), however, current barriers in mass cultivation of microalgae are limiting its successful industrialization. Therefore, a mathematical model integrating both the biological and hydrodynamical parts of the cultivation process may improve our understanding of relevant phenomena, leading to further optimization of the microalgae cultivation.

**Results:**

We introduce a unified multidisciplinary simulation tool for microalgae culture systems, particularly the photobioreactors. Our approach describes changes of cell growth determined by dynamics of heterogeneous environmental conditions such as irradiation and mixing of the culture. Presented framework consists of (i) a simplified model of microalgae growth in a culture system (the advection-diffusion-reaction system within a phenomenological model of photosynthesis and photoinhibition), (ii) the fluid dynamics (Navier-Stokes equations), and (iii) the irradiance field description (Beer-Lambert law). To validate the method, a simple case study leading to hydrodynamically induced fluctuating light conditions was chosen. The integration of computational fluid dynamics (ANSYS Fluent) revealed the inner property of the system, the flashing light enhancement phenomenon, known from experiments.

**Conclusion:**

Our physically accurate model of microalgae culture naturally exhibits features of real system, can be applied to any geometry of microalgae mass cultivation and thus is suitable for biotechnological applications.

## Background

The global warming, gradual oil fuel depletion, higher demands for energy and food consumptions are some of the current challenges our society is facing. Thus, looking for alternative resources is a hot topic. After the failure of the first generation biofuels based on corn and soya, which created a food shortage in the third world, the scientific community focused on simple photosynthetic organisms, cyanobacteria and microalgae. These organisms have high photosynthetic efficiency, biomass growth and lipids content [1].

Cyanobacteria and microalgae are very versatile microorganisms which have attracted a significant amount of scientific interest in the last decades [2–4]. These unicellular oxygenic organisms absorb photons of specific wavelengths to fix inorganic carbon (CO_2_). Carbon molecules are then utilized in the Calvin-Benson cycle, producing complex organic molecules in dark photosynthetic reactions and thus reducing the concentration of carbon dioxide in the environment. In addition, cyanobacteria and microalgae consume inorganic phosphorus and nitrogen which could be used in the processes of waste-water treatment [5]. The current scientific focus is to employ microalgae and cyanobacteria in biotechnology field as a potential biofuel source, for example a genetic modification increasing lipid content [6]. The other applications, e.g., pharmaceutical (bioactive metabolites), nutritional (animal feed and human food and supplements), are less important compared to the renewable energy business.

However, there are technological and knowledge-based barriers preventing optimized mass cultivation of photosynthetic microorganisms. In particular, reliable approaches allowing qualitative predictions for simulation of microbial biomass production in microalgae culture systems (MCS), including the photobioreactors (PBR), are rather slowly emerging than being well established [7–9]. The cause of apparent lack of accurate models of microalgae growth is in the complexity of three-dimensional multiphase (liquid-gas-solid) flow dynamics, irradiance distribution within MCS and multi-level processes of cellular functions. A reliable model has to integrate these phenomena which interact across various time-scales. Therefore, it is necessary to solve theoretical (multi-time-scale phenomena) and practical (high computational requirements) problems. As a result, the complex interactions within the MCS are often integrated in inadequate way, leading to over-simplified models [10]. Especially, the correct integration of a computational fluid dynamics (CFD) code and photosynthetic reaction kinetics is essential for meaningful solution [11]. Finally, current models, describing general MCS or specific PBR performance analysis, have usually no connection to the bio-production itself, e.g., [4, 12], and references within.

In this study, we present a unified modeling framework of fluid dynamics and photosynthetic reaction kinetics for microalgae culture system of any geometry. The framework is validated by a numerical simulation of microalgae growth in a 2-D square cavity with one moving wall, a hypothetical system triggering a vortex flow. This system is able to reproduce the hydrodynamically induced high frequency light-dark cycles regime causing the so-called flashing light enhancement [13–15].

## Methods

We present a simulation tool for MCS (photobioreactor, open or raceway ponds) to connect the key aspect of real system, i.e., microalgae cell growth under changing hydrodynamical and optical conditions. This framework consists of (i) a state system – mass balance equations in form of advection-diffusion-reaction partial differential equations – PDEs (1-3), (ii) the fluid flow equations, i.e., Navier-Stokes equations (4), and (iii) the irradiance distribution inside MCS according to Beer-Lambert’s law (5), see [8, 16].

All mentioned parts of the introduced MCS model are interconnected, i.e., the mass balance equations for the state variables (species characteristics, nutrients, gases, etc) are solved simultaneously with the fluid dynamics (momentum balances, continuity equations). Nevertheless, we assume that the stationary flow field inside the MCS is not affected by mass transfer and reactions [17]. This assumption allows the detachment of biological and environmental states and thus biological and environmental parts (models) can be analyzed with different numerical methods and with different spatial-temporal discretizations, dramatically reducing the computational demands. This detachment could be complete (for the stationary flow field in a continuous system) or stepwise, e.g., reflecting some sequence of quasi-steady states in a production system operated in batch mode.

### State model

The biological part of modeling framework is based on the mass balance equations for the state variables describing the transport and reaction among the species or compounds [18]:

1 $$ \begin{aligned} \frac{\partial c_{i}}{\partial t} + \nabla \cdot ({v}c_{i}) - \nabla \cdot (D_{e}\nabla c_{i}) = R(c_{i}) + S(c_{i})~,\\ ~ t \in [\!t_{0},T],~ i=1,\ldots,m,  \end{aligned}  $$

where *c*_*i*_=*c*_*i*_(*x*,*t*), is a conservative quantity (concentration or cell density), *v* is a velocity of flow field ruled by the fluid-dynamic model, cf. (4), and *x*∈*Ω*⊂*R*^3^ stands for a position vector in a coordinate system (e.g., Cartesian or cylindrical). The dispersion coefficient *D*_*e*_ is a second-order tensor, which corresponds to the diffusion coefficient in microstructure description. *D*_*e*_ is an empirical parameter describing mixing in the system and it is influenced by the molecular diffusion and velocity profile, i.e., it is not strictly a material constant.

The reaction kinetics and temporal changes of the reacting species are described by the reaction term *R*(*c*_*i*_) and source terms *S*(*c*_*i*_), respectively. The source term, e.g., the external load of nutrients into MCS, is usually modeled as a corresponding boundary condition. However, in order to (i) simplify the analytic study of the optimal solution existence, see [19], and (ii) respect that the location of discharge of some material could be inside the domain *Ω*, we employed the particular form of (1). The initial condition and boundary condition (impermeability of the domain boundary, e.g., PBR walls) are following:

2 $$ ~ c_{i_{0}} = c_{i}(x,t_{0}), ~x \in \Omega \subset R^{3}, ~ i=1,\ldots,m,   $$

3 $$ ~\nabla c_{i}(x, t) = 0,~ x \in \partial \Omega, ~ t \in [t_{0},T],~ i=1,\ldots,m.   $$

### Fluid-dynamic model

Microalgal cells are considered to be solid particles and fixed CO_2_ and released O_2_ are gases, therefore the analyzed system can be described as a multiphase flow and transport. However, it is possible to consider our multiphase system as a suspension by neglecting the gaseous phase. Then, the microalgal cells represent the dispersed phase of a suspension. Also, considering the cell density about 10 kg m ^−3^ for the dry weight of biomass, i.e., 1% of mass content, allows us to classify our system as a single-phase system based on Newtonian viscosity relationship within the employed computational software ANSYS Fluent [20]. Moreover, we assume that the mean diameter of a spherical microalgae cell is approximately ten micrometers [5] which supports this simplification.

Mass density of the suspension is determined as *ρ*=*ρ*_*w*_ (1−*k*)+*ρ*_*s*_ *k*, where *ρ*_*w*_ is the mass density of the medium and *ρ*_*s*_ represents the cell mass density, and *k* is the volume fraction. However, one can assume *ρ*=*ρ*_*w*_ because of a homogeneous distribution of algal cells (no aggregation) which are actually floating in the medium. Furthermore, the inter-particle distances in our case of dilute suspension are sufficiently large to calculate flow field over each particle or cell [18], i.e., particles do not interfere with the flow field. This idea has been proposed by J. Pruvost et al. [21] who showed that the smallest eddy size, based on Kolmogorov scale, is approximately ten times bigger than the cell size, i.e., neglecting the cell-vortex interaction.

Therefore, we model the incompressible liquid phase (suspension of water, nutrients and microalgae), thus the classical system of Navier-Stokes equations and the continuity equation is employed as:

4 $$ \frac{\partial v}{\partial t} + (v \cdot \nabla) {v} = f - \frac{1}{\rho}\nabla p + \nu \nabla^{2} v, ~~~ \nabla \cdot v = 0,   $$

in [ *t*_0_,*T*],×*Ω*, with suitable boundary conditions on [ *t*_0_,*T*],×*∂Ω* and initial conditions in *Ω*. *v*,*p*,*f*,*ρ* and *ν* denote the fluid velocity, the pressure, the body forces, fluid density and kinematic viscosity, respectively.

### Reaction model and its time-scale analysis

The photosynthetic reactions depend on many variables such as irradiance, accessibility of both inorganic (Fe, P, etc) and organic nutrients (e.g., carbohydrates), concentration of gases, osmolality, and many others. In this work, we focus only on the key variable determining the reaction kinetics, the fluctuations of irradiance; the other variables are assumed to be externally controlled to not limit the cellular growth. Generally, both light attenuation and scattering should be considered to describe the irradiance distribution inside MCS [16]. Moreover, for complicated MCS geometries and multiple light sources distribution, the irradiance field has to be determined experimentally. Here, for the sake of simplicity and manageable computational costs, we suppose the Beer-Lambert law (5) is valid, i.e., the incident irradiance *I*_0_ is exponentially decreasing with the increasing light path *s*:

5 $$ I(s) = I_{0} ~e^{-\Lambda s},   $$

where *Λ* is an attenuation coefficient describing the attenuation of irradiance by the suspension of microbial cells in the liquid medium (unit: m ^−1^).

Based on the decades of experimental research of photosynthesis, the reliable model of reaction kinetics in MCS should implement no less than three time scales across the photosynthetic reactions, see [22]. The following systems are integrated in the reaction term *R*(*c*_*i*_) in (1): (i) activation of the light harvesting complex (photosynthetic unit – PSU) in light reactions, (ii) biomass output, and (iii) photoinhibition, i.e., damage of pigment-protein complexes by excessive irradiance. Photoacclimation (light adaptation) is another important process which, however, can be observed in the tens of hours-days time scale. Therefore, it is possible to model it as a (quasi) steady state mechanism, e.g., implicitly via a reaction model parameter when another differential equation describes dynamics of this parameter [23].

Comparison of the characteristic times of microalgae growth (∼ hours) with the mixing due to dispersion – turbulent diffusion (∼ seconds) indicates a huge gap between both processes. There is a possibility to adopt a very simple growth modeling approach (e.g., Monod or Haldane kinetics) leading to two alternatives. The first one is governed by the time scale of chosen steady state kinetics and has to neglect details concerning mixing phenomena. The second alternative, based on the time step in order of seconds, can observe transitions because of the hydrodynamic mixing but has to neglect the changes in photosynthetic growth (in form of a growth kinetic model). However, both alternatives entirely miss the connection across the transport and reaction processes.

As a suitable candidate for the reaction model, we adopted a mechanistic three-state model of photosynthetic factory (PSF) [24–29]. PSF model considers 3 states in which microalgae cells may exist: *activated – A, inhibited – B* and *rested – R*. Although the fluid-dynamical properties of cells in each of three states are exactly the same, these states are impacted differently by dynamic changes of the environment, i.e., spatio-temporal changes of states concentrations determine the biomass production, see (6).

Let be the concentrations of respective components *c*_*A*_, *c*_*B*_, and *c*_*R*_ (identical units as for the microalgae cell density *c*_*x*_ in whole PBR – generally 10^6^ cell ml ^−1^ as in [28]). Then the following relation holds (for ∀*t*∈[*t*_0_,*t*_*∞*_], and ∀*x*∈*∂Ω*): *c*_*A*_(*x*,*t*)+*c*_*B*_(*x*,*t*)+*c*_*R*_(*x*,*t*)=*c*_*x*_(*x*,*t*). The dimensionless scalars *y*_*A*_=*c*_*A*_/*c*_*x*_, *y*_*B*_=*c*_*B*_/*c*_*x*_ and *y*_*R*_=*c*_*R*_/*c*_*x*_ (molar fractions) are respective states of the PSF model, see the next section.

We note that the spatio-temporal averaged rate of photosynthetic production (specific growth rate $\mu := \dot {c_{x}}/c_{x}$) is related (constant *κ*) to the activated state fraction [28, 30]:

6 $$ \dot c_{x} =\frac{\kappa \gamma}{\text{meas}(\Omega) ~T} \int_{0}^{T} \int_{\Omega}^{}\left [{y_{A}(I(x),t) - Me}\right ] c_{x} ~\mathrm{d}x~ \mathrm{d}t~,  $$

where term *Me* represents a cellular maintenance; *Me* is considered constant although it may vary if hydrodynamical shear stress is considered [29].

Decimal value 10^−4^ [s ^−1^] of term *κγ* in (6) allows the shift from the time scale of light fluctuation (caused by fluid dynamics or the light source via the irradiance distribution *I*(*x*) in PSF model) to the time scale of biomass growth (macro-scale) with no impact on the accuracy. Then, the integral in (6) is evaluated exactly and factor *κγ* in (6) provides the value of a specific growth rate. Thus, the employed PSF model satisfies the requirement for the time scales of the model candidate. Three of its five parameters can be seen as time constants for three different time scales: i) the light and dark reactions (∼ seconds), ii) the photoinhibition (∼ minutes) and iii) the microalgae growth (∼ hours). The remaining two parameters represent the optimal irradiance and the conversion from dimensionless growth to the real values, see [27, 31] for more details.

### Model of photosynthetic factory – PSF model

**Fig. 1:**
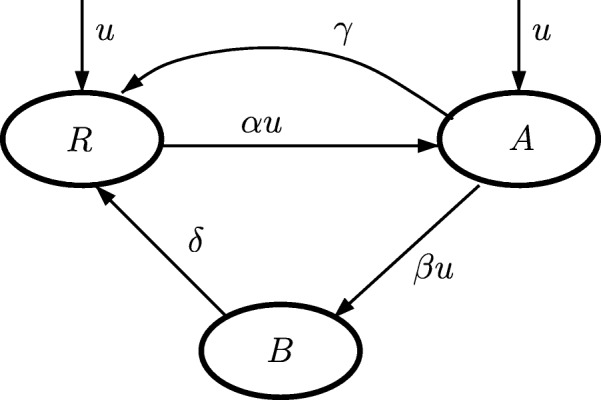
Scheme of Photosynthetic factory model (PSF). Four parameters (*α*, *β*, *γ*, *δ*) describe transitions among respective states (resting *R*, activated *A*, and *B* inhibited)

Here we characterize the multi time-scale three-state model of photosynthetic factory (PSF model) proposed by Eilers and Peeters [24, 25] and further developed by Wu and Merchuk [28, 29] and Celikovsky, Papacek and Rehak [26, 27, 30–32]. PSF model (Fig. 1), is used for derivation of the reaction term *R*(*c*_*i*_), see the transport Eq. (1).

PSF model (7) incorporates the dynamics of three fundamental states propagating in different orders of time scales: (i) cell growth (including the shear stress effect), (ii) photoinhibition, and (iii) photosynthetic light and dark reactions. The state vector of the PSF model is three dimensional, *y*=(*y*_*R*_,*y*_*A*_,*y*_*B*_)^⊤^, where *y*_*R*_ indicates the probability that PSF is in the resting state *R*, *y*_*A*_ the probability that PSF is in the activated state *A*, and *y*_*B*_ the probability that PSF is in the inhibited state *B*.

7 $$  \begin{array}{c} \left[ \begin{array}{c} \dot{y_{R}} \\ \dot {y_{A}} \\ \dot {y_{B}} \end{array} \right] \,=\, \left[ \begin{array}{rrr} 0 & \gamma & \delta \\ 0 & - \gamma & 0 \\ 0 & 0 & - \delta \end{array} \right] \left[ \begin{array}{c} y_{R} \\ {y_{A}} \\ {y_{B}} \end{array} \right]\\ \qquad \qquad\qquad \,\,\,\,+ u(t)\left[ \begin{array}{rrr} - \alpha & 0 & 0 \\ \alpha & - \beta & 0 \\ 0 & \beta & 0 \end{array} \right] \left[ \begin{array}{c} y_{R} \\ {y_{A}} \\ {y_{B}} \end{array} \right] \end{array}  $$

The values of PSF model parameters, i.e., *α*, *β*, *γ*, *δ*, see (7), and *κ*, see (6), are taken from original study [28], where Wu and Merchuk identified these values of PSF model parameters for the microalga *Porphyridium* sp.: *α* = 1.935 × 10^−3^ *μE*^−1^ m^2^, *β* = 5.785 × 10^−7^ *μE*^−1^ m^2^, *γ* = 1.460 × 10^−1^ s ^−1^, *δ* = 4.796 × 10 ^−4^ s ^−1^, *κ* = 3.647 10 ^−3^ and *Me* = 0.059 h ^−1^. For details regarding the experimental setup, for parameter estimation and the identifiability study, see [31, 32].

In order to make the PSF model parameter estimation more robust, the following re-parametrization was introduced and the singular perturbation method was used in [31]: $q_{1}: = \sqrt {\frac { \gamma \delta }{ \alpha \beta }}, ~ q_{2}: = \sqrt {\frac {\alpha \beta \gamma }{\delta {(\alpha +\beta)}^{2}}},~ q_{3} := \kappa \gamma \sqrt {\frac { \alpha \delta }{\beta \gamma }},~ q_{4}:=\alpha q_{1},~ q_{5} := \beta /\alpha.$ Consequently, the PSF model acquires the following form:

8 $$ \dot{y} = \left[ {\mathcal{A}}+u(t){\mathcal{B}} \right] y,  $$

9 $$ {\mathcal{A}}=q_{4} \left[ \begin{array}{ccc} 0 & {q_{2}(1+{q_{5}})} & \frac{q_{5}}{q_{2}(1+{q_{5}})} \\ 0 & - {q_{2}(1+{q_{5}})} & 0 \\ 0 & 0 & - \frac{q_{5}}{q_{2}(1+{q_{5}})} \end{array} \right],  $$

10 $$ ~~ {\mathcal B}=q_{4} \left[ \begin{array}{ccc} - 1 & 0 & 0 \\ 1 & - q_{5} & 0 \\ 0 & q_{5} & 0 \end{array} \right].  $$

For the given input variable, i.e., the irradiance *u*(*t*), the ODE system (7) can be solved either by numerical methods or by asymptotic methods. However, the irradiance level of 250 *μ*E m ^−2^ s ^−1^ (the value of the parameter *q*_1_ which maximizes the steady-state growth [31]), provides two negative eigenvalues of the (7) system matrix: *λ*_1_ = -0.63, *λ*_2_ = -0.59 10 ^−3^. The ODE system (7) is *stiff* due to value 10^3^ of the ratio $\frac {\lambda _{1}}{\lambda _{2}}$. This fact points to the existence of two processes: (i) photosynthetic reactions, and (ii) photoinhibition, widely separated from the point of view of their characteristic times. Further, without significant loss of accuracy, we use the so-called fast reduction [27], i.e., the behavior of the system (7) is characterized by the only one following ODE

11 $$  \begin{array}{c} \frac{\mathrm{d} y_{A}}{\mathrm{d}t} = - q_{4} \left(u_{av} + q_{2}\right)\left[\frac{u(z) + q_{2}}{u_{av} + q_{2}}y_{A} - \frac{u(z)}{u_{av}} {y_{A}}_{ss}(u_{av})~\right], \end{array}  $$

and the “slow” variable *y*_*B*_ can be regarded as a constant depending on the averaged value *u*=*u*_*av*_. According to our works [27, 33] it holds

12 $$ \begin{array}{c} {{y_{A}}_{ss}(u)} = \frac{u/q_{2}}{ u^{2}+u/q_{2}+1}, \\ ~{{y_{B}}_{ss}(u)} = \frac{u^{2}}{ u^{2}+u/q_{2}+1} ~.  \end{array}  $$

Both the non-reduced (7) and the reduced order PSF model Eq. (11) have been incorporated as a User-Defined Function (UDF) in ANSYS Fluent, see the following section.

### Numerical aspects of PDEs implementation and solution

The key issue is in the incorporation of a CFD code and photosynthetic reaction kinetics in one modeling framework. A proper analysis of characteristic times of microalgae growth and of mixing due to the convective motion is especially important. Two appealing approaches, Lattice Boltzmann method [34] and hybrid multicompartment/CFD approach [35], were previously used for bioreactor modeling [36, 37]. Both methods seem to be computationally efficient, however, the discussion of their advantages and disadvantages is far of the scope of this paper. Our approach is based on the implementation of a User-Defined Function (UDF) within the commercial CFD code ANSYS Fluent, which provides the possibility to define an arbitrary reaction term, see below.

As denoted in the previous section, ODE system (7) can be simplified to only one differential Eq. (11). The right-hand part of this equation indicates the rate of change of activated state *y*_*A*_ which can be used in the definition of UDF function in ANSYS Fluent. Macro DEFINE_VR_RATE provides the possibility to define the arbitrary reaction term in (1), including its dependency on the spatial coordinate as represented by (5). Macro C_CENTROID can retrieve the corresponding coordinates of the current mesh cell. Three states of the microalgae culture were represented as individual species with the same molar weights in ANSYS Fluent. Therefore, mass and molar fractions should be identical. Example of the user-defined reaction rate definition employed in our simulations for the reduced system (11) follows:


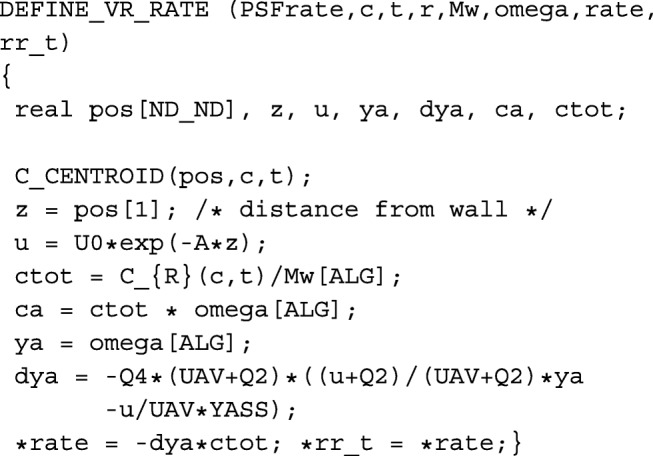


**Fig. 2:**
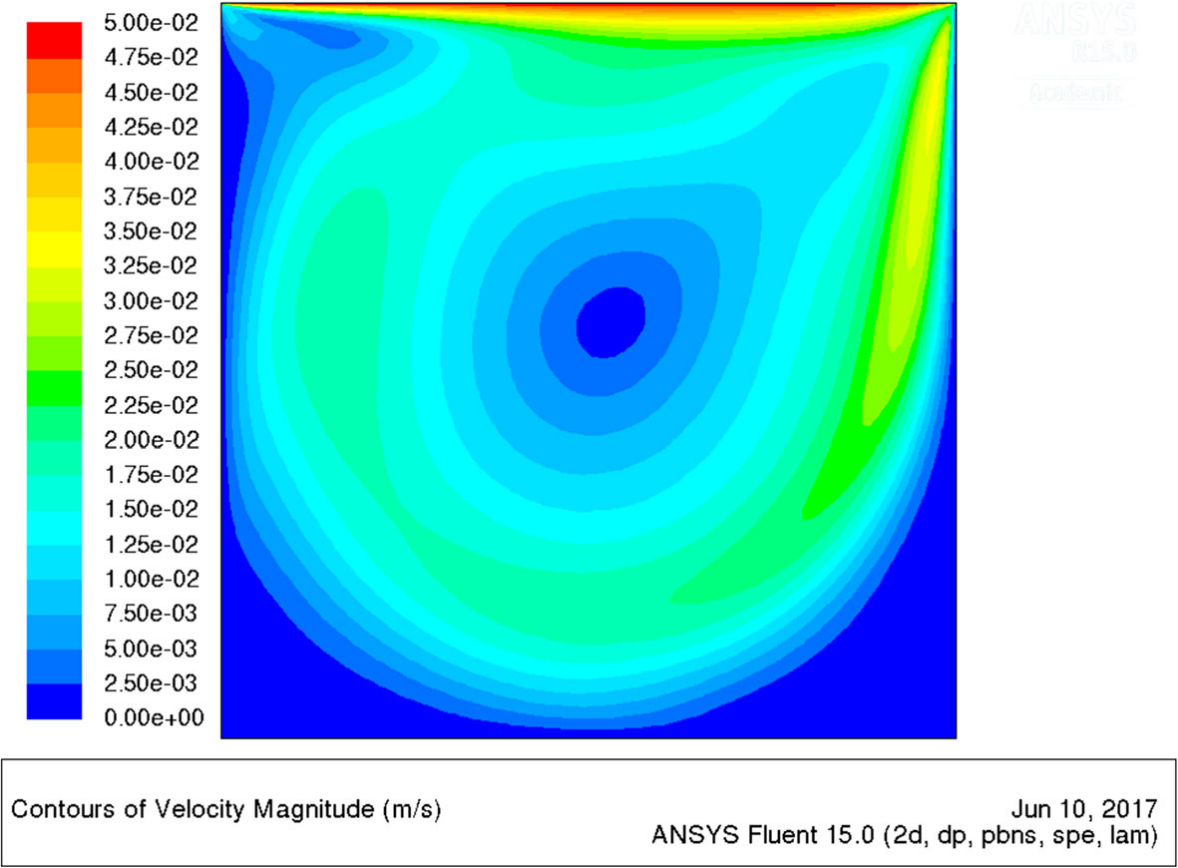
Contours of steady-state velocity magnitude of the microalgae suspension within the idealized 2-D square cavity. The vortex flow is imposed by the moving upper wall (wall coordinate is *L* and its velocity is *v*_*L*_ in *x*-direction, i.e., from left to right), corresponding Reynolds number is Re=1000 in this case (laminar flow regime)

We performed a grid independence study for three mesh sizes of the given geometry (2-D square cavity with moving wall), see Fig. 2. Grid Convergence Index (GCI) describing an average velocity of the flow field for the finest mesh was evaluated as 1.14% according to Celik et al. [38]. All simulations of the 2-D geometry were performed on a grid with approx. 10 thousand mesh elements. Refined mesh elements near walls were created so that dimensionless thickness *Y*^+^ was around 1 to capture properly the viscous sublayer in the turbulent flow regime. Time step 0.025 s was used and the accuracy of the solution at the end of time range 0 – 5 s was evaluated as 0.19%.

Every simulation consisted of two steps. In the first step, the Navier-Stokes Eq. (4) were solved iteratively to get a converged solution of the steady-state flow field for the corresponding mixing rate (Reynolds number). In the second step, the flow field was assumed to be fully developed and only Eq. (1) describing the transport and reaction of individual species (states of the algae in our case) were solved. This approach is more efficient but in cases when the flow field exhibits a transient behaviour, the Navier-Stokes equations must be solved simultaneously with the species transport equations. For example, the Coutte-Taylor device shows an inherently transient behaviour at some specific rotations rates [39]. Periodic oscillations can be observed even in the square cavity [40, 41] which we neglected in this study.

## Results and discussion

**Fig. 3:**
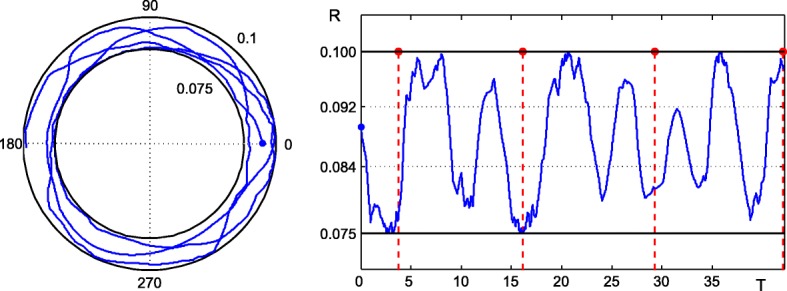
Result of CFD simulation of one particle trajectory by CFD code ANSYS Fluent [20]. The particle trajectory in the Couette-Taylor device cross-section is shown on the left side. The right-side picture describes the time course of the microalgae particle radial position *R*. The “irradiance history” of an individual cell can be calculated by concatenation of trajectory and the irradiance field *I*=*f*(*R*,*t*) within the device

Our early works [27, 30] theoretically confirmed the phenomenon of flashing light enhancement, known from experiments [13–15]. However, these models were applicable only for specific PBR geometries, being a Couette-Taylor bioreactor in [30] and flat panel PBR in [27], and suffered from over-simplification (Navier-Stokes equations were not solved there within a fluid dynamic model). Here, for the first time, we introduce a general approach applicable to any geometry of MCS and thus ready for industrial applications. For the validation of our integrated model, we have chosen the special geometry consisting of a 2-D square cavity with one moving wall (Fig. 2), representing an idealized part of a Couette-Taylor device, see Fig. 3. We note that microalgae cells are forced to travel between dark and light sides of the Couette-Taylor device (see Taylor vortex in [39]) which is an analogy to the flashing light regime and consequently the flashing light enhancement [15]. Indeed, very high cell densities (about 150 g/l) were reported in this type of PBR by E. A. Davis in [42]. The author claims that the growth enhancement is due to an ordered mixing (E. A. Davis misuses the term “turbulence”) produced by the Couette-Taylor device. Nevertheless, the mixing enhanced light and mass transfer is only one part of the whole picture. Based on the fact that the standing issue of microalgae mass production in PBR is the cell fragility [43], we see also the other enhancing effect - low shear stress transmitted to microbial cells in the laminar Taylor vortex flow regime [44].

### Photosynthesis in fluctuating light regime

The growth of photosynthetic microorganisms could be described by the steady-state photosynthetic reaction kinetics called *P–I (photosynthesis-irradiance) curve* representing the photosynthetic response in small cultivation systems with a homogeneous light distribution [17]. However, MCS models based on *P–I* curves only do not take into account the effect of hydrodynamic mixing (or mixing via static mixers) which has a different characteristic time scale compared to the time scale of the steady-state reaction kinetics, see “Reaction model and its time-scale analysis” section and [27, 31] for more details. In order to overcome this problem, Terry in [15] employed “artificial” corrections to the steady-state kinetic model reflecting the enhancement in the photosynthetic efficiency during fluctuating light conditions present in the dynamic model.

In our work, we aim to build a general predictive model, independent of specific experimental data. Such model must be built on universal principle that time-scales of all relevant processes are considered; see details in section “Reaction model and its time-scale analysis”.

**Fig. 4:**
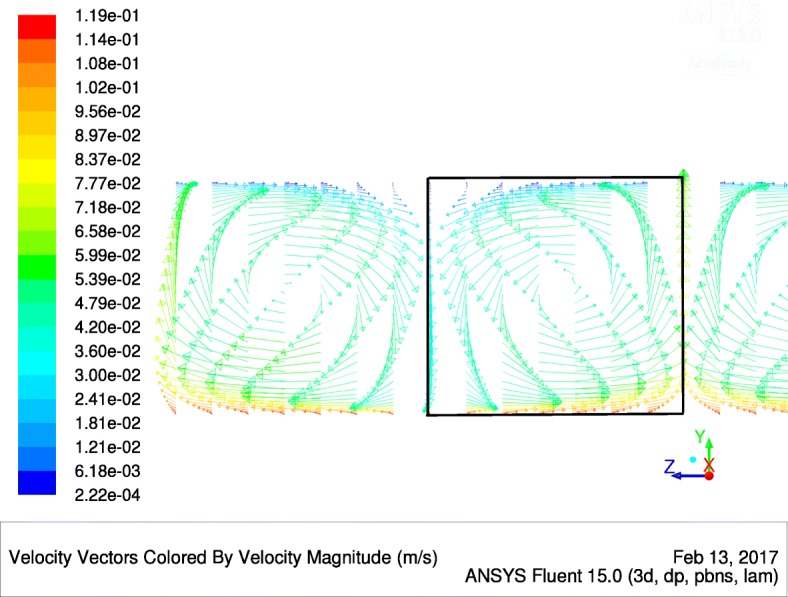
Fluid flow velocity profile in the axial section of the laboratory Couette-Taylor device calculated by CFD code ANSYS Fluent 15.0 (laminar model, inner cylinder angular frequency *ω* = 2.4 rad s ^−1^, Re=2000). One rectangular cell used for the 2D case study is marked by the bold rectangle

To determine if our modeling framework is able to describe the flashing light enhancement phenomenon, we have chosen a 2-D square cavity with the moving top wall as our hypothetical MCS, see Fig. 2. This MCS can be viewed as the representation of a Couette-Taylor device, i.e., one cell from its axial cross-section when the so-called Taylor vortex flow regime takes place. This occurs when the Taylor number exceeds the first critical value [39], see Fig. 4. The cultivation system is illuminated from the bottom side only by the incident irradiance *I*_0_ and the irradiance level is exponentially decreasing with the increasing z-coordinate until the top (*z*=*L*) according to the Beer-Lambert law (5). It is convenient to work further with the normalized irradiance *u*=*I*/*I*_*opt*_, where *I*_*opt*_ reaches the value of 250 *μ*E m ^−2^ s ^−1^ [28]. Our analysis showed that the value of the averaged irradiance within MCS *u*_*av*_=1, see Table 1, provides an optimal condition for the microalgae culture growth. This optimization problem depends mainly on mixing intensity and it is discussed elsewhere [33]. Only for the lumped parameter system, the value *u*=1 is the optimal one. The relations for the irradiance level in depth *z* and for the average (absorbed) irradiance in the whole MCS are then:

13 $$ u(z) = u_{0} ~e^{-\Lambda z}~,~ u_{av} = u_{0}\frac{1 - e^{-\Lambda L}}{\Lambda L}~,   $$

**Table 1:** Basic parameters specifying the growth rate in MCS system used in this study and CFD simulations: *u*_*av*_ represents the value of average irradiance in the culture, *L* stands for MCS characteristic length, velocity of the upper MCS wall is given by Reynolds number Re, *ΛL* stands for optical thickness, *q*_2_, *q*_4_, *y*_*R*_(*t*_0_), *y*_*A*_(*t*_0_) and *y*_*B*__*ss*_(*u*_*av*_=1) represent 2 parameters, 2 initial conditions for rested and activated state of the PSF model and (quasi) steady state level of the inhibited state, respectively (see section “Model of photosynthetic factory – PSF model”)

*u*_*av*_	*L* [m]	Re =*v*_*L*_ *L*/*ν*	*Λ* *L*	*q*_2_	*q*_4_ [*s*^−1^]	*y*_*R*_(*t*_0_)	*y*_*A*_(*t*_0_)	*y*_*B*__*ss*_(*u*_*av*_)
1	0.02	0 – 100000	8 ln(2)	0.3	0.5	1	0	0.1875

where *u*_0_=*I*_0_/*I*_*opt*_ is the incident irradiance on the bottom (*z*=0) and *L* is the MCS thickness in direction of light gradient. It is further convenient to define a dimensionless “optical thickness” of the system *Ot*>0 as follows: *Ot*:=*ΛL*. It holds $\frac {r_{1/2}}{L}=\frac {\ln (2)}{Ot}$, where *r*_1/2_ is the length interval (unit: m) in which the light intensity diminishes to one half. Then, ratio $\frac {u(z)}{u_{av}}$ for optically thin culture system (*Ot*≈5.5 in our case) has

14 $$\frac{u(z)}{u_{av}}\approx {\Lambda L}~{e^{-\Lambda L \frac{z}{L}}}.  $$

We suggest that the “irradiance history” of an individual cell within our system in Fig. 2 corresponds to the hydrodynamically induced light/dark cycles shown in Fig. 3 (produced by the Couette-Taylor device). The irradiance history can be calculated by linking the trajectory of an individual microalgae cell and the irradiance field within device.

The final step is the implementation of the transport-reaction model based on the PDEs (1-4). The key role plays the reaction term *R*(*c*_*i*_) in (1). The *R*(*c*_*i*_) term is “local” in sense that it depends on the actual transport and reaction rates, and it contains all relevant time scales, thus, it perfectly fits to our requirements. The technical simplicity of PDE based approach, which takes advantage of the sophisticated CFD codes, e.g., ANSYS Fluent [20], is obvious when compared to the alternative Lagrangian approach. While the Eulerian approach reaches its governing equations from the mass balance in an infinitesimal control volume, the Lagrangian approach is based on the above mentioned irradiance history of an individual cell. Although conceptually simple, the application of Lagrangian approach to MCS modeling presents serious problems. First, either an experimental technique, e.g., computer-automated radioactive particle tracking (CARPT) [45] or a CFD code is needed for the cell trajectory description, see Fig. 3. Second, using an ensemble of cell trajectories across the irradiance field inside MCS, the probabilistic description of “irradiance history” of individual cells is reached. Finally, the problem reduces to a reaction kinetics model represented by an ordinary differential equation with a stochastic input.

The employed Eulerian approach is more convenient, mainly because it benefits from CFD codes and the implementation of an appropriate dynamic model of microbial growth (with lumped parameters) into a distributed parameter system is merely technical.

### Case study: Biological performance of MCS in flashing-light regime

Having the modeling framework assembled, we aimed to reproduce the hydrodynamic regime where high frequency light-dark cycles are induced and trigger the flashing light enhancement [14, 15]. The values of parameters employed in the simulation of MCS performance are summarized in Table 1.

The PSF model consists of the ODE system (7), nevertheless it can be reduced by using the singular perturbation method [33]. Then, the behavior of the system (7) is characterized by only one ODE (11). Furthermore, for the local description of PSF model, we need the expression for *u*(*z*) in the case of average irradiance *u*_*av*_=1; based on (14): $u(z)\approx \Lambda L~{e^{-\Lambda L \frac {z}{L}}}$.

**Fig. 5:**
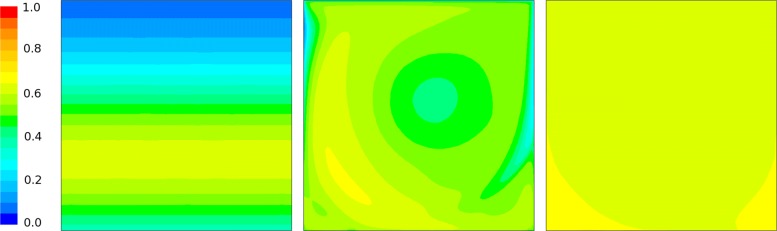
The steady-state spatial distribution of the activated state molar fraction *y*_*A*_ for three different Reynolds numbers, Re = 0, 1000, 100000, from left to right. The irradiation, governed by the Lambert-Beer law (13), is maximal on the bottom side. Contour plots of the state *y*_*A*_ were calculated by the CFD code ANSYS Fluent 15.0 using the non-reduced (full) PSF model (7). The dynamics of *y*_*A*_ state (and *y*_*B*_ as well) is depicted in Fig. 8

**Fig. 6:**
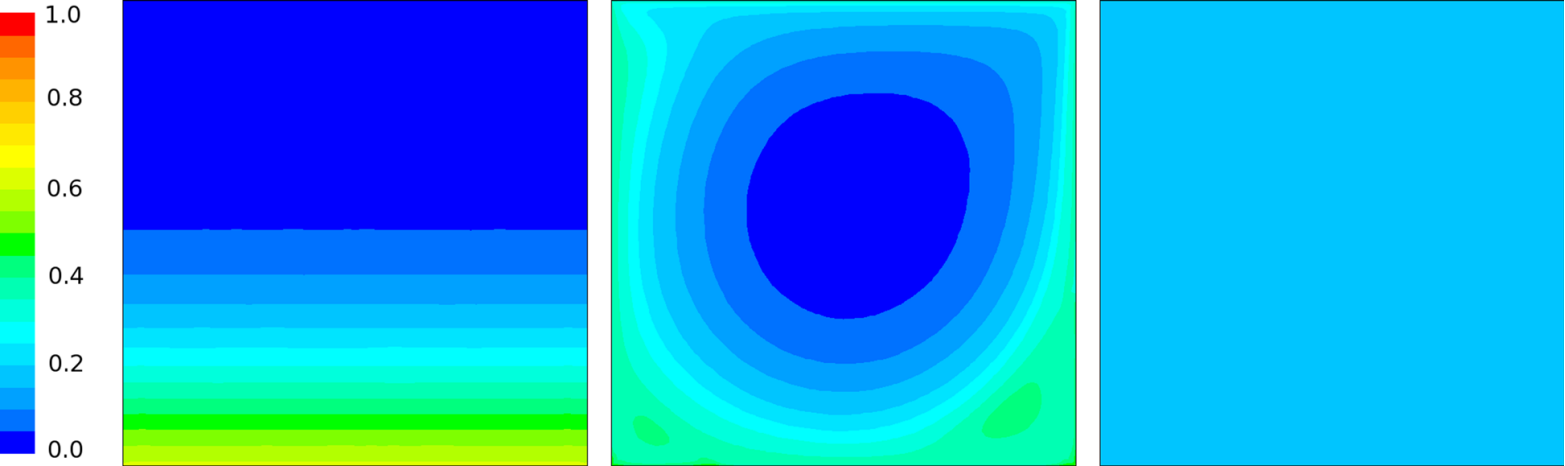
Contour plots of the inhibited state *y*_*B*_ calculated by the CFD code ANSYS Fluent 15.0 using the non-reduced model (7) for different mixing rates. The steady-state spatial distribution of *y*_*B*_ shows the clear pattern for low mixing conditions corresponding to no mixing (Left: Re = 0) and low mixing (Center: Re = 1000), while for an almost ideally mixed case (Right: Re = 100000) the *y*_*B*_ concentration is evenly distributed within the reaction domain. The numerical values are clearly consistent with the results shown on the right part of Fig. 8 (non-reduced model)

**Fig. 7:**
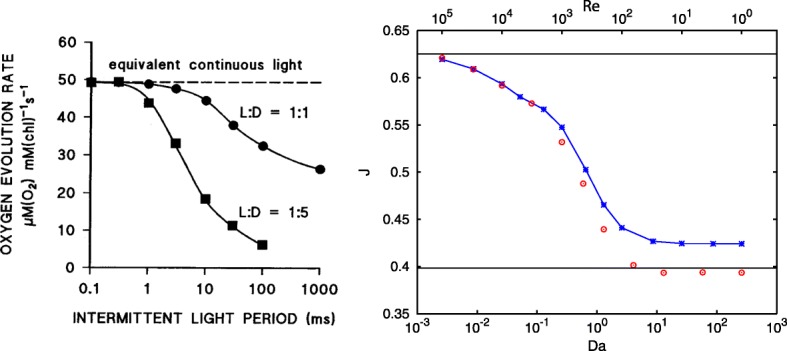
Simulated and experimental results of the flashing light enhancement phenomenon. Left picture shows experimental data reprinted from [14]. Two curves of *Oxygen evolution rate vs. L-D cycles period* are tagged with the ratio of light-to-dark interval L:D. Right: the normalized performance index *J* vs. Da, for larger mixing rate (i.e., lower Da - bigger Re), we get better performance *J* approaching its maximal theoretical value corresponding to the growth in averaged continuous light, cf. (12). Red circles represents simulation results of the whole non-reduced system (7) or (8) gained for time range 0 - 20000 s, with time step 0.1 s

**Fig. 8:**
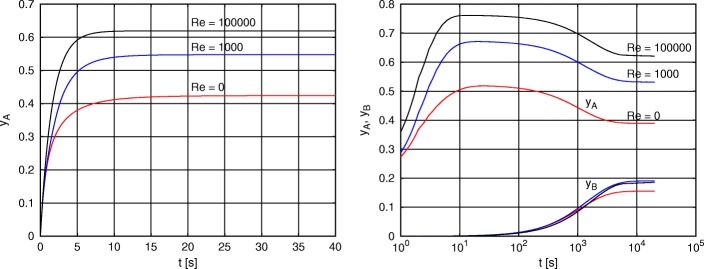
Time dependency of the activated state *y*_*A*_ for three different mixing intensities, Re = 0, 1000, 100000, with and without fast reduction, respectively. On the left, the time dependency of activated state *y*_*A*_ of the reduced system (11) is shown for time range 0 - 40 s. On the right, the time dependency of activated state *y*_*A*_ and inhibited state *y*_*B*_ of the non-reduced system (7) is shown for time range 0 - 20000 s. The dynamics of activated state *y*_*A*_ has two phases, fast phase (the transition from the initial value *y*_*A*_(*t*_0_)=0 to the maximal value occurring in seconds) and slow phase (imposed by the gradually growing inhibited state *y*_*B*_). In the case of applied fast reduction (left graph), the slow dynamics does not occur due to constant

For the initial condition and other parameters in Table 1, the case study was resolved using CFD code ANSYS Fluent: PDE (1-4) embedded and the reaction kinetics implemented as a special UDF (User-Defined n) according to the description in section “Numerical aspects of PDEs implementation and solution”. The resulting steady-state solution for molar fraction *y*_*A*_ is shown in Fig. 5. The spatial distribution of molar fraction *y*_*B*_ steady-state solution is shown in Fig. 6. The spatio-temporal average for molar fraction *y*_*A*_ is shown in Fig. 7 and, finally, the temporal evolution of *y*_*B*_ steady-state is depicted in Fig. 8 (right). Notice nice patterns of *y*_*B*_ for low mixing conditions in Fig. 6 (left and center).

It is clear that the presented simulations are in the qualitative match with the previously published experimental data [14], see Fig. 7 on the left. Also, the time scale of *y*_*A*_ state dynamics is shown in Fig. 8, describing the time course of the spatial average of *y*_*A*_ for three different velocities *v*_*L*_, corresponding to Reynolds number of 0, 1000, and 100000, respectively. The mixing intensity determines the frequency of hydrodynamically induced light/dark cycles. Similarly to our previous simplified studies [27, 30], we can see the hydrodynamically induced growth enhancement due to the flashing light. The major difference and improvement to previous studies is that presented modeling framework can be used to any MCS setup and not only to special cases.

Analysis of our numerical model indicates that the process is characterized by the *Damköhler number* Da:=*t*_*tr*_/*t*_*r*_ which describes the ratio between the characteristic time of transport ($t_{tr}=\frac {L}{v_{L}}$) and reaction time (*t*_*r*_=1/[*q*_4_(*u*_*av*_+*q*_2_)]). In chemical engineering, the Damköhler numbers Da are frequently used to describe the ratio between the chemical reaction time scale (reaction rate) and the flow time scale (convective mass transport rate) occurring in a system. For our specific case, when the characteristic time *t*_*r*_ is fixed, it holds:

$${\text{Da}}:=1/t_{r} \frac{L^{2}}{\nu}\frac{1}{\text{Re}}=\frac{260}{\text{Re}}. $$

It is known that extremely high or low values of the characteristic numbers leads to numerical difficulties during solving PDEs, e.g., (1). Employing Monod or Haldane kinetics (steady-state) might lead to such difficulties and that is why a multi time-scale model would be more appropriate.

In order to quantify the impact of mixing velocity on the cellular growth, we defined an objective function *J*_*MCS*_ as a volumetric productivity (usually in grams per liter per day):

15 $$  J_{MCS} = \frac{1}{\text{meas}(\Omega) ~T} \int_{0}^{T} \int_{\Omega}^{}\left[{y_{A}(u,z,t)}\right] c_{x} ~\mathrm{d}z \mathrm{d}t~.  $$

For a usual case of steady state operation, it is only the activated state fraction *y*_*A*_(*u*,*z*,*t*) in *J*_*MCS*_ which has to be integrated over the MCS domain. Thus, in Fig. 7 we show the dependence of the normalized performance index

$$\frac{J}{c_{x}}=\frac{1}{L^{2}}\int_{\Omega}^{}{y_{A}(u,z,t)} ~\mathrm{d}z ~. $$

We note that the empirical data have an illustrative and testing purpose only and for quantitative simulations one has to employ parameters for specific MCS and particular microalgae. However, the result of biological performance for our MCS compared to experimental results shown in Fig. 7 (left) clearly shows that the positive impact of mixing on the growth induced by the convective motion, i.e., the simulated flashing light enhancement is comparable to experiments. Analyzing simulations in Fig. 7 (right) implies that lower Da (bigger *v*, better mixing) leads to better performance *J* (approaching its maximal theoretical value 0.625). Nevertheless, the harmful impact of the hydrodynamically induced shear stress originally considered in *Me* term [29], see (6), is not taken into account and thus in reality certain mixing intensity would start damaging the cellular integrity.

## Conclusions

In order to reach commercially sustainable production for algae biofuels, the empirical data indicates the need for accurate and reliable modeling approach with both predictive and optimizing capabilities. In this work, we presented the general unified modeling framework for microalgae culture systems (MCS) which can be applied to an arbitrary geometry of the production system and any microbial strain in general. This allows evaluating the performance of various MCS architectures, operating conditions, and microalgae strains as well. All parts of the multidisciplinary framework, i.e., the growth-rate model, the fluid-dynamic model, and the irradiance-distribution model, are integrated within CFD package ANSYS Fluent. In the illustrative case study, we have shown that a three-state model of photosynthetic factory (PSF model) well behaves under hydrodynamically induced high frequency light-dark cycles regime and copes with the requirement imposed on the reaction model, i.e., it correctly describes both the quasi steady-state (flashing light enhancement) and dynamic phenomena (time course of the PSF model activated state).

We can conclude that our modeling framework provides an adequate and physically accurate description of microalgae growth in a MCS and thus it is suited for the optimal control problem formulation as well. To the extent of our knowledge, this is the first time when such advanced solution was successfully used in biotechnology of microalgae.

Our future plans are to further focus on the optimization problem and its formulation. Finally, we plan to analyze the shear stress impact on the microalgae growth.

## Abbreviations

CARPT: Computer-automated radioactive particle tracking
CFD: Computational fluid dynamics
GCI: Grid convergence index
MCS: Microalgae culture systems
PBR: Photobioreactors
PDEs: Partial differential equations
PI: Photosynthesis-irradiance
PSF: Photosynthetic factory
UDF: User-defined function
